# LncRNA SNHG10 is downregulated in non-small cell lung cancer and predicts poor survival

**DOI:** 10.1186/s12890-020-01281-w

**Published:** 2020-10-20

**Authors:** Meng Liang, Linlin Wang, Chuanhua Cao, Shimao Song, Feng Wu

**Affiliations:** 1 Department of Oncology Taihe Hospital, Hubei University of Medicine, 32 South Renmin Road, Shiyan, Hubei 442000 People’s Republic of China; 2 Department of Radiology, Renmin Hospital, Hubei University of Medicine, Shiyan, Hubei 442000 People’s Republic of China; 3 Department of Oncology, Xiangyang Central Hospital, Affiliated Hospital of Hubei University of Arts and Science, Xiangyang, Hubei 441021 People’s Republic of China

**Keywords:** SNHG10, miR-21, NSCLC, Methylation

## Abstract

**Background:**

LncRNA SNHG10 has been reported to be an oncogenic lncRNA in liver cancer. However, its roles in non-small cell lung cancer (NSCLC) remains unknown.

**Methods:**

Tumor and paired non-tumor tissues were harvested from 62 NSCLC patients. RT-qPCR was used to detect the expression of SNHG10 and miR-21 in tissues. Overexpression experiments were used to evaluate the interaction between SNHG10 and miR-21 in NSCLC cells. CCK-8 assay was used to detect the cell proliferation.

**Results:**

We observed the expression of SNHG10 was down-regulated in non-small cell lung cancer (NSCLC) compared with that in non-tumor tissues. Moreover, we found that high expression levels of SNHG10 predicted favorable survival of NSCLC patients, and the expression of miR-21 were increased in NSCLC and inversely correlated with SNHG10 expression. In NSCLC cells, overexpression of SNHG10 resulted in increased miR-21 gene methylation and decreased miR-21 expression. Moreover, overexpression of SNHG10 attenuated the enhancing effect of miR-21 overexpression on cell proliferation.

**Conclusions:**

SNHG10 may involve in NSCLC cell proliferation by regulating the miR-21 gene methylation.

## Background

Non-small cell lung cancer (NSCLC) is the major subtype of lung cancer and is the main cause of cancer-related deaths worldwide [1]. NSCLC has two major subtypes including lung squamous cell carcinoma (LUSC) and lung adenocarcinoma (LUAD) [2]. Despite of the advances have been made on the treatment and diagnosis of NSCLC, only less than 15% of NSCLC patients can survive for more than 5 years [3]. Therefore, more effective therapeutic approaches are needed. Smoking is the major risk factor for NSCLC [4]. However, never-smokers also develop NSCLC [5], suggesting the involvement of other factors, such as genetic factors, in the molecular pathogenesis of NSCLC [6].

It has been well established that molecular players participate in nearly all aspects of the occurrence and development of NSCLC [7, 8]. Increased understanding of the molecular mechanism of NSCLC provides novel targets for the development of anti-cancer approaches, such as targeted therapy [9, 10]. LncRNAs are emerging critical players in cancer biology and they participate in cancer mainly by regulating the expression of cancer-related genes [11, 12]. Therefore, lncRNAs are potential targets for cancer targeted therapy [13]. SNHG10 has been characterized as an oncogenic lncRNA in liver cancer [14]. However, we observed the downregulation of SNHG10 in NSCLC and it’s inversely correlation with miR-21 by exploring the TCGA dataset. It is known that miR-21 is a key oncogenic miRNA in cancer [15]. This study was therefore performed to investigate the role of SNHG10 and miR-21in NSCLC.

## Methods

### Patients and follow-up

This study enrolled a total of 62 NSCLC patients (30 cases of LUAD and 32 cases of LUSC) between May 2013 and January 2015 at Taihe Hospital, and was approved by the Ethics Committee of Taihe hospital. All patients were confirmed by histopathological biopsy, and no patients received any therapy for any clinical disorders within 3 months before this study. Other severe clinical disorders were excluded from these patients. Based on AJCC staging system, there were 28 cases at stage I or II, and 34 cases at stage III or IV. Informed consent was signed by all patients. From the day of admission, the 62 patients were followed up for 5 years. The patients were visited every month through phone call. Patients died of non-NSCLC were excluded from this study. The follow-up was completed by all patients.

### Tissue collection

All patients were subjected to biopsy prior to therapy. During biopsy, NSCLC and paired non-tumor tissues were obtained from each patient. Histopathological exam was used to confirm all of the collected tissues. In addition, tissues were immediately subjected to RNA extraction after collections.

### Cell culture and transfection

To match the patients included in this study, the LUSC cell line KLN 205 and LUAD cell line HCC827 were used. RPMI-1640 medium (90%) and FBS (10%) were used to conduct the cell culture. A 5% CO_2_ incubator was used to cultivate both cell lines at 37 °C. SNHG10 expressing vector was constructed using pcDNA3.1 (Invitrogen) as the backbone vector. Mimic of miR-21 and negative control (NC) miRNA were purchased from Sigma-Aldrich. Vectors (1 μg) or miRNAs (40 nM) were transfected into KLN 205 and HCC827 cells (1 × 10^8^) using lipofectamine 2000 (Invitrogen). KLN 205 and HCC827 cells (1 × 10^8^) were transfected with either NC miRNA or empty vector to serve as NC group. Cells were cultivated for further 48 h prior to the following experiments.

### RT-qPCR

Isolation of RNA from tissues and in vitro cultured cells was performed using Ribozol (Invitrogen). DNase I was used to incubate with RNA samples at 37 °C for 2 h to completely digest genomic DNA. RNA samples were reverse transcribed into cDNA samples using a Reverse Transcription System (A5001, Promega Corporation). With cDNA samples as template, qPCRs were carried out to determine the expression of SNHG10 using SYBR Green Master Mix (Bio-Rad). The internal control of SNHG10 was 18S rRNA. Addition of poly (A) was added to mature miRNAs, following by miRNA reverse transcriptions and miRNA qPCRs to determine the expression of miR-21, and the endogenous control for miR-21 was U6. Three replicates were set for each experiment and Ct values were calculated using the 2^-ΔΔCT^ method.

### Methylation-Specific PCR (MSP)

After transfected with empty vector or SNHG10 expression vector, KLN 205 and HCC827 cells were used to extract genomic DNAs using Genomic DNA Extraction Kit (ab156900, Abcam). DNA samples were converted using DNA Methylation-Gold™ kit (ZYMO RESEARCH). After that, the methylation of miR-21 was evaluated by Taq 2X master mix (NEB).

### Cell Counting Kit-8 (CCK-8) assay

After transfected with empty vector or SNHG10 expression vector, KLN 205 and HCC827 cells were subjected to cell proliferation analysis using CCK-8 kit (Dojindo). Cells were washed with ice-cold PBS, followed by cell counting. After that, 3000 cells in 0.1 ml medium were transferred to each well of a 96-well plate, followed by cell culture at 37 °C. OD values (450 nm) were measured every 24 h for a total of 4 d. At 4 h before the measurement of OD values, CCK-8 solution was added into each well to reach 10%.

### Statistical analysis

Mean ± SD values were used in this study. Difference between two groups was evaluated by paired *t-*test. Differences among multiple groups were analyzed by ANOVA (one way) and followed by Tukey’s test. Linear regression was used to evaluate the correlations. The 62 patients were divided into high and low SNHG10 level groups (*n* = 31, cutoff value was the median expression level of SNHG10 in NSCLC tissues) to analyze survival. K-M method and Log-rank test were used to plot and analyze the survival curves. *P* < 0.05 was considered as statistically significant.

## Results

### Downregulation of SNHG10 is correlated with the poor survival of NSCLC patients

**Fig. 1:**
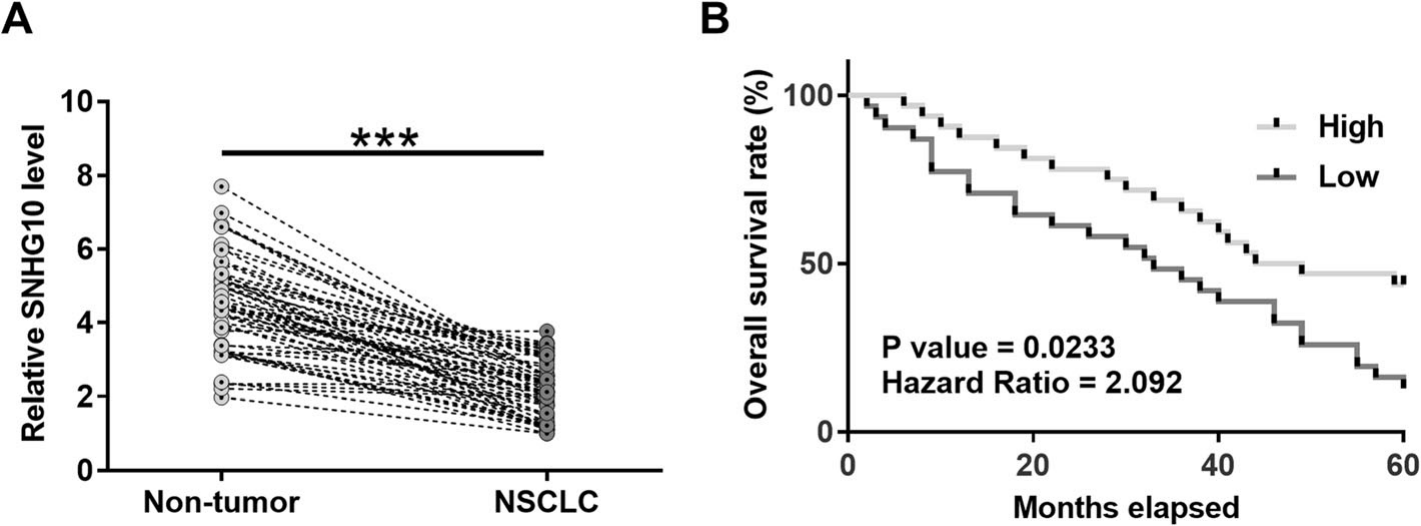
Downregulation of SNHG10 is correlated with the poor survival of NSCLC patients. Expression of SNHG10 in paired tissues was determined by RT-qPCR. Levels of SNHG10 expression were compared between NSCLC and non-tumor tissues. Mean values were compared (**a**). ***, *p* < 0.001. To analyze survival, the 62 patients were divided into high and low SNHG10 level groups (*n* = 31, with median expression level of SNHG10 in NSCLC tissues as cutoff value). Survival curves were plotted and compared by log-rank test (**b**)

In order to investigate the expression of SNHG10 in NSCLC patients, TCGA dataset was used to analyze the expression of SNHG10. The result showed that SNHG10 was downregulated in both LUAD (4.04 vs. 8.20) and LUSC (5.71 vs. 8.29) in comparison to that in tumor tissues. To further confirm the downregulation of SNHG10 in NSCLC, the expression of SNHG10 in NSCLC tissues and its paired non-tumor tissues was evaluated by RT-qPCR. Compared with non-tumor tissues, NSCLC tissues exhibited significantly lower expression levels of SNHG10 (Fig. 1a, *p* < 0.001). Survival curve analysis revealed that patients in high SNHG10 level group showed higher overall survival rate compared to patients in low SNHG10 level group (Fig. 1b).

### MiR-21 was upregulated in NSCLC and inversely correlated with SNHG10

**Fig. 2:**
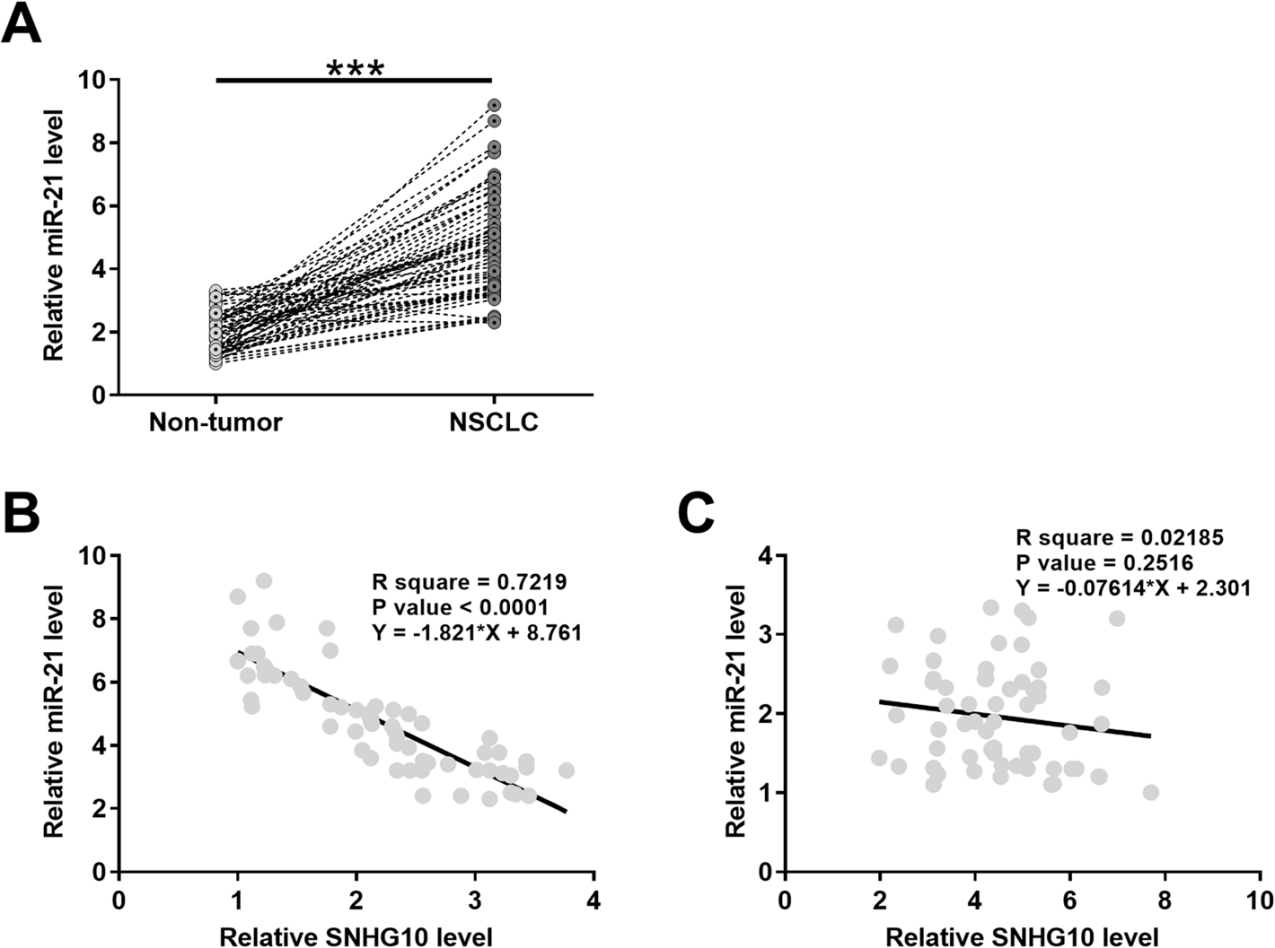
MiR-21 was upregulated in NSCLC and inversely correlated with SNHG10. Expression of miR-21 in paired NSCLC and non-tumor tissues from the 62 patients was determined by RT-qPCR. Levels of miR-21 expression were compared between NSCLC and non-tumor tissues. Mean values were compared (**a**). ***, *p* < 0.001. Linear regression was performed to analyze the correlations between SNHG10 and miR-21 across NSCLC tissues (**b**) and non-tumor tissues (**c**)

RT-qPCR was used to evaluate the miR-21 expression in paired NSCLC and non-tumor tissues. The results showed that miR-21 expression was significantly increased in NSCLC tissues compared with non-tumor tissues (Fig. 2a, *p* < 0.001). Moreover, the expression levels of miR-21 and SNHG10 were inversely and significantly correlated across NSCLC tissues (Fig. 2b), but not across non-tumor tissues (Fig. 2c) evaluated by correlation analysis.

### SNHG10 downregulated miR-21 in NSCLC cell through methylation

**Fig. 3:**
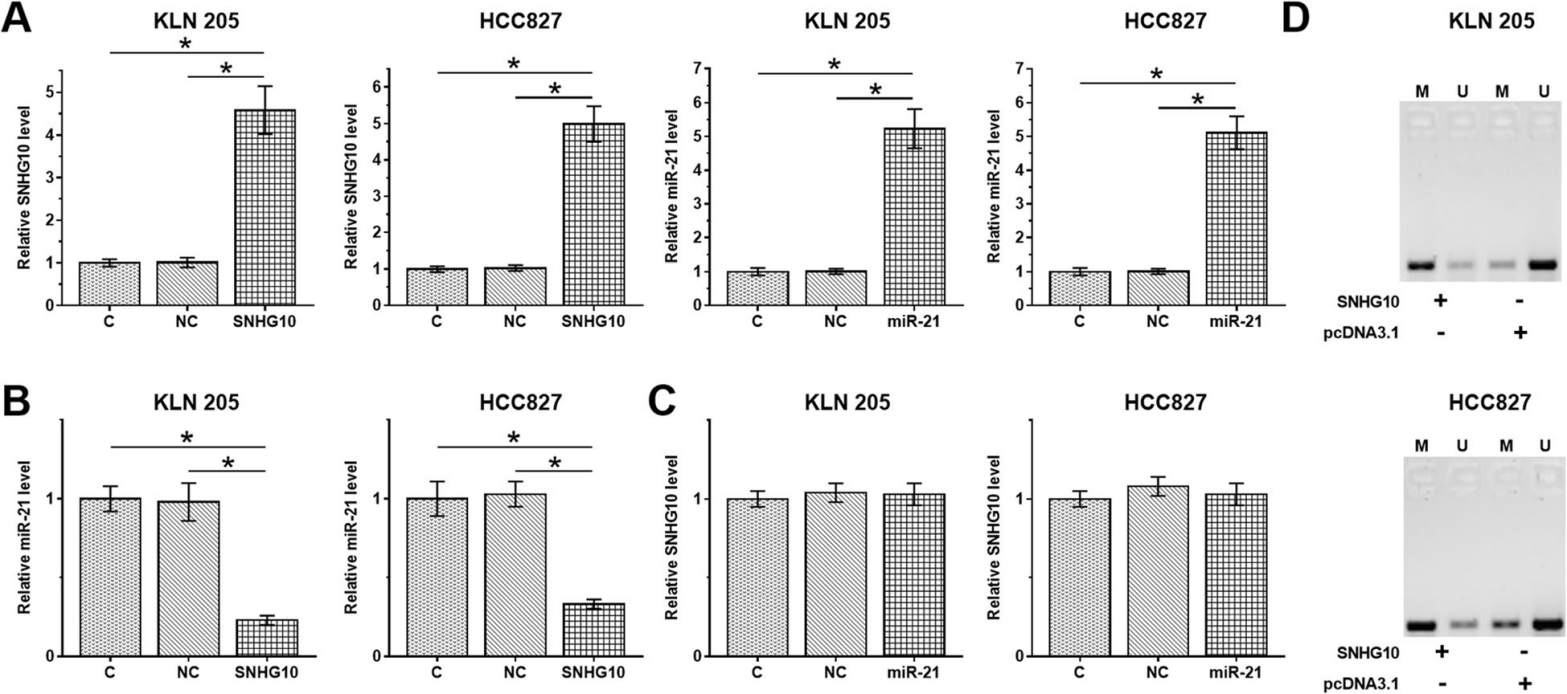
SNHG10 downregulated miR-21 in NSCLC cell through methylation. SNHG10 expression vector or miR-21 mimic was transfected into KLN 205 and HCC827 cells. Transfections were confirmed by RT-qPCR (**a**). The effects of SNHG10 overexpression on miR-21 (**b**), and the effects of miR-21 overexpression on SNHG10 (**c**) were also analyzed by RT-qPCR. MSP was performed to analyze the effects of SNHG10 overexpression on miR-21 (**d**). Mean ± SD values were presented and compared. M, methylation; U, un-methylation; *, *p* < 0.05.

KLN 205 and HCC827 cells were used to overexpress the SNHG10 or miR-21, and RT-qPCR was used to confirm these overexpression (Fig. 3a, *p* < 0.05). The results showed that SNHG10 overexpression resulted in downregulation of miR-21 expression in KLN 205 and HCC827 cells (Fig. 3b, *p* < 0.05), while miR-21 overexpression did not affect the SNHG10 expression in KLN 205 and HCC827 cells (Fig. 3c, *p* > 0.05). The effects of SNHG10 overexpression on miR-21 methylation was evaluated by MSP. In addition, SNHG10 overexpression showed significantly increased miR-21 methylation (Fig. 3d).

### Overexpression of SNHG10 attenuated the enhancing effect of miR-21 overexpression on cell proliferation

**Fig. 4:**
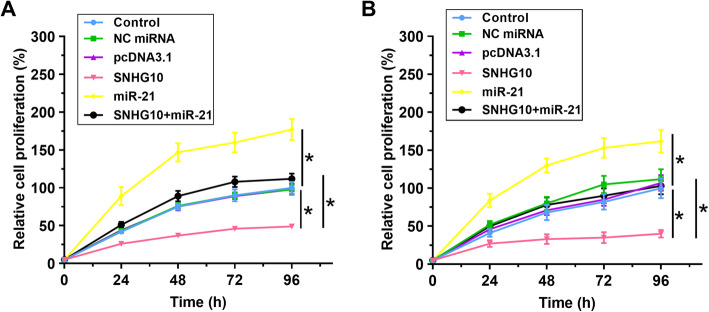
SNHG10 overexpression attenuated the enhancing effect of miR-21 overexpression on cell proliferation. The roles of SNHG10 and miR-21 in regulating the proliferation of KLN 205 and HCC827 cells were analyzed by CCK-8. Mean ± SD values were presented and compared. *, *p* < 0.05

The roles of SNHG10 and miR-21 in regulating the proliferation of KLN 205 and HCC827 cells were evaluated by CCK-8. Compared with control cells, decreased proliferation of cells was observed after the overexpression of SNHG10, while increased proliferation of cells was observed after the overexpression of miR-21. Moreover, overexpression of SNHG10 attenuated the enhancing effect of miR-21 overexpression on cell proliferation (Fig. 4, *p* < 0.05).

## Discussion

In the present study, we aimed to investigate the role and underlying mechanism of SNHG10 in NSCLC. Clinical data showed that SNHG10 was downregulated in NSCLC and predicted poor survival of NSCLC patients. Additionally, miR-21 was up-regulated and negatively correlated with SHG10 in NSCLC. In two NSCLC cell lines, we revealed that SNHG10 reduced miR-21 via methylation. Moreover, SNHG10 inhibited the proliferation of NSCLC cells by targeting miR-21. Therefore, SNHG10 is a tumor suppressor in NSCLC.

lncRNAs have been found to be involved in the development of cancer. For example, lncRNA DANCR could enhance cancer cell migration and invasion in gastric cancer [16]. LncRNA XIST could induce proliferation in pancreatic cancer cells [17]. A recent study reported that SNHG10 was an oncogenic lncRNA in liver cancer. It is reported that SNHG10 was upregulated in liver cancer and formed a positive feedback loop with its homolog SCARNA13, thereby promoting cancer metastasis [14]. Interestingly, SNHG10 was remarkably downregulated in NSCLC according to our analyses of TCGA dataset. We confirmed this finding by determining the expression of SNHG10 in paired NSCLC and non-tumor tissues. In two NSCLC cell lines, overexpression of SNHG10 resulted in decreased proliferation of NSCLC cells. Therefore, SNHG10 is likely a tumor suppressor lncRNA in NSCLC, and SNHG10 may play different roles in different types of cancer, suggesting that NSCLC and liver cancer may have different molecular pathogenesis.

Even with active treatments, such as surgical resection and chemotherapy, the overall survival of NSCLC is still poor [18, 19]. In this study, we showed that overexpression of SNHG10 suppressed the proliferation of NSCLC, and high expression levels of SNHG10 were correlated with the favorable survival of NSCLC patients. Therefore, SNHG10 may serve as a target for the treatment of NSCLC. In addition, measuring the expression levels of SNHG10 before therapy may assist the prognosis of NSCLC, thereby guiding the determination of treatments and improve patients’ survival.

MiR-21 is a well-characterized oncogenic miRNA that promotes tumorigenesis in many cancers, such as cervical, breast and gastric cancers [20–22]. In NSCLC, miR-21 is a serum biomarker for detection of early-stage NSCLC, and has been found to enhance cancer progression by targeting its targets genes, like SOCS1, PTEN, SOX7 [23–25]. However, the upstream regulators of miR-21 have not been well studied. In this study, we found that miR-21 was negatively correlated with SNHG10 in NSCLC tissues. Moreover, SNHG10 was directly regulated by SNHG10 through methylation, and it was involved in the inhibitory effect of SNHG10 on NSCLC cell proliferation. Hence, SNHG10 is an upstream regulator of miR-21 and can inhibits its oncogenic function in NSCLC. It is worth noting that SNHG10 and miR-21 were only closely correlated across NSCLC tissues, but not across non-tumor tissues. Therefore, certain pathological factors may mediate the interaction between them, and further studies are needed.

## Conclusion

In conclusion, SNHG10 is downregulated, and miR-21 was upregulated in NSCLC. SNHG10 predicts the prognosis of NSCLC, and it can downregulate miR-21 through methylation to suppress the proliferation of cancer cells.

## Data Availability

All data generated or analysed during this study are included in this published article. The data that support the findings of this study are available from Taihe Hospital, Hubei University of Medicine but restrictions apply to the availability of these data, which were used under license for the current study, and so are not publicly available. Data are however available from the authors upon reasonable request and with permission of Taihe Hospital, Hubei University of Medicine.

## Abbreviations

NSCLC: Non-small cell lung cancer
LUSC: Lung squamous cell carcinoma
LUAD: Lung adenocarcinoma
MSP: Methylation-specific PCR
CCK-8: Cell Counting Kit-8 assay
